# Low Health Literacy in Patients with Steatotic Liver Disease and Depressive Symptoms

**DOI:** 10.3390/jcm15218474

**Published:** 2026-10-30

**Authors:** Leonard Kaps, Christian Labenz, Alexander Barton, Jörn M. Schattenberg, Maurice Michel

**Affiliations:** 1 Department of Dermatology, University Medical Center of the Johannes Gutenberg University Mainz, 55131 Mainz, Germany; 2 Department of Medicine II, Saarland University Medical Center, Saarland University, 66421 Homburg, Germany; alexander.barton@uks.eu (A.B.); joern.schattenberg@uks.eu (J.M.S.); 3 First Department of Medicine, University Medical Center of the Johannes Gutenberg University Mainz, 55131 Mainz, Germany; christian.labenz@unimedizin-mainz.de; 4 Cirrhosis Center Mainz (CCM), University Medical Center of the Johannes Gutenberg University Mainz, 55131 Mainz, Germany

**Keywords:** health literacy, steatotic liver disease, MASLD, depression, Health Literacy Questionnaire, liver stiffness

## Abstract

**Background and Objectives:** Health literacy (HL) affects the course of chronic diseases, yet little is known about HL in steatotic liver disease (SLD), and the role of mental comorbidities has not been examined. This study assessed HL in patients with SLD and sought to identify factors linked to lower HL. **Materials and Methods:** This bicentric cross-sectional study comprised 234 patients with SLD: 209 recruited prospectively in Homburg, Germany (2024–2025), and 25 patients with metabolic dysfunction-associated steatohepatitis (MASH)-related cirrhosis from an existing cohort in Mainz. HL was measured with the German version of the nine-scale Health Literacy Questionnaire (HLQ). SLD was defined by a controlled attenuation parameter ≥ 275 dB/m, and advanced chronic liver disease (ACLD) by liver stiffness ≥ 15 kPa (Baveno VII/VIII) or known cirrhosis. Depressive symptoms were assessed with the Patient Health Questionnaire-9 (PHQ-9 ≥ 10; Homburg) or the Hamilton Depression Rating Scale (HDRS-17 ≥ 8; Mainz). **Results:** Sixty-eight patients (29%) had depressive symptoms (Homburg 25.8%, Mainz 56.0%). The groups were comparable with respect to age, sex, schooling, liver stiffness and BMI. In univariable analyses, patients with depressive symptoms had lower scores on scales 2, 3, 6 and 7. After adjustment for sex, age, BMI, schooling, and ACLD, associations remained significant for scale 2 (“Having sufficient information to manage my health”; β = −0.14, *p* = 0.04), scale 3 (“Actively managing my health”; β = −0.15, *p* = 0.02), scale 6 (“Ability to actively engage with healthcare providers”; β = −0.21, *p* = 0.003) and scale 7 (“Navigating the healthcare system”; β = −0.16, *p* = 0.02). The association with scale 6 was consistent across all sensitivity analyses, including an analysis restricted to the Homburg cohort. ACLD was not independently associated with any HLQ scale, whereas higher BMI was associated with lower scores in five of nine domains. **Conclusions:** More than one in four patients with SLD has depressive symptoms, which are associated with lower self-reported HL, especially regarding engagement with healthcare providers and navigation of the healthcare system. Screening for depression could help to identify patients who require additional support.

## 1. Introduction

Modern healthcare has become increasingly complex, and many patients, particularly those living with chronic conditions, find it hard to move through care pathways and to manage their illness effectively [1,2]. Against this background, health literacy (HL) is understood as a concept that covers not only a person’s ability to deal with health-related information but also the obligation of healthcare systems to make their services accessible [3]. In the health promotion glossary of the World Health Organization (WHO), HL is described as the set of cognitive and social skills that shape an individual’s motivation and capacity to find, understand and make use of information for the benefit of their health [4].

Low HL has repeatedly been linked to unfavorable outcomes, especially in people with chronic illness. Affected patients are admitted to hospital more often, cause higher healthcare expenditure and have a higher risk of death; they also make less use of preventive services, attend scheduled follow-up visits less reliably and adhere less well to their medication. Furthermore, limited HL hampers communication between patients and healthcare professionals and goes along with a poorer understanding of the underlying disease [5,6,7,8,9,10,11,12].

Patients with chronic diseases may be particularly affected by limited HL. This applies especially to patients with liver cirrhosis, who often additionally experience neurocognitive deficits, for instance, as a consequence of hepatic encephalopathy [13]. In a direct comparison with other chronic conditions such as inflammatory bowel disease or even gastrointestinal malignancies, patients with cirrhosis showed a particularly high susceptibility to low HL [14]. Whereas cirrhosis is the common end point of chronic liver diseases, metabolic dysfunction-associated steatotic liver disease (MASLD) is now the most rapidly increasing liver disease worldwide, fueled by physical inactivity and excessive caloric intake. According to the current multisociety nomenclature, MASLD requires hepatic steatosis (steatotic liver disease, SLD; fat in ≥ 5% of hepatocytes) in combination with at least one cardiometabolic risk factor, i.e., overweight or obesity, type 2 diabetes mellitus, dyslipidemia, arterial hypertension or insulin resistance, provided that other relevant causes of liver fat accumulation have been excluded [15]. In addition to its metabolic sequelae, MASLD has repeatedly been linked to an increased prevalence of depression [16]. Data from several large cohorts in different countries point to a reciprocal relationship: depression may predispose to MASLD, and MASLD may in turn promote depression, which could adversely affect the clinical course [17,18,19].

The main objective of this bicentric study was therefore to examine, by means of the multidimensional and German-validated Health Literacy Questionnaire (HLQ), whether depressive symptoms are related to lower HL in patients with SLD. Our hypothesis was that depressive symptoms would show an independent association with lower HL scores.

## 2. Materials and Methods

This bicentric analysis comprised a total of 234 patients with SLD (Supplementary Figure S1). Of these, 209 were enrolled prospectively from August 2024 to August 2025 in the outpatient clinic of the Department of Medicine II, Saarland University Medical Center, Homburg, Germany. A further 25 patients with SLD were drawn retrospectively from an existing cohort in Mainz for this exploratory analysis. This cohort had been recruited in 2019 and 2020 from the liver outpatient clinic and the general wards of the First Department of Medicine, University Medical Center of the Johannes Gutenberg University Mainz. Among the 89 patients with cirrhosis of any etiology in this cohort who had completed the HLQ, all 25 with metabolic dysfunction-associated steatohepatitis (MASH)-related cirrhosis were selected [13].

All patients underwent a detailed medical history covering social and disease-related aspects. In Homburg, daily alcohol consumption was assessed in a structured interview, and patients additionally completed the Alcohol Use Disorders Identification Test (AUDIT). Patients reporting excessive alcohol intake (≥20 g/day for women and ≥30 g/day for men, i.e., metabolic dysfunction and alcohol-associated liver disease [MetALD] or alcohol-related liver disease according to the multisociety nomenclature [15]) were not invited to participate; the same applied to patients with other established causes of chronic liver disease, such as viral hepatitis or autoimmune or cholestatic liver disease. SLD was diagnosed when the controlled attenuation parameter (CAP; FibroScan^®^) was ≥275 dB/m, and cardiometabolic risk factors were documented according to the MASLD criteria [15]. Following the Baveno VII criteria and the recently published Baveno VIII consensus [20,21], a liver stiffness measurement (LSM) < 10 kPa was regarded as excluding compensated advanced chronic liver disease (cACLD), values between 10 and 15 kPa as suggestive and values ≥ 15 kPa as highly suggestive of cACLD. In the analyses, advanced chronic liver disease (ACLD) was defined as LSM ≥ 15 kPa. Patients in the grey zone (LSM 10–15 kPa) were counted as not having ACLD in the regression models and were omitted from the stratified comparison (see Section 3.3); a sensitivity analysis applying a threshold of ≥10 kPa was performed in addition. LSM was available for the Homburg cohort only. All Mainz patients had cirrhosis due to MASH (previously termed non-alcoholic steatohepatitis, NASH), which had been diagnosed by the treating hepatologists on the basis of clinical, laboratory, imaging and/or histological findings after other etiologies, including alcohol-related liver disease, had been ruled out; these patients were therefore classified as having ACLD. As this group included patients with both compensated and decompensated disease, the combined variable is referred to as ACLD rather than cACLD. In line with Baveno VII/VIII, decompensation was defined as current or past clinically overt ascites, variceal hemorrhage and/or overt hepatic encephalopathy [20,21].

The two centers used different instruments to assess depressive symptoms. In Mainz, where the cohort had originally been set up under a separate protocol for patients with cirrhosis, depressive symptoms were rated by clinicians with the 17-item Hamilton Depression Rating Scale (HDRS-17). In Homburg, the self-administered Patient Health Questionnaire-9 (PHQ-9) was selected because it is short and practicable as a screening tool in routine outpatient care. For the PHQ-9, a score ≥ 10 was used as the cut-off; this threshold corresponds to at least moderate depressive symptoms and offers the best balance of sensitivity and specificity for major depression [22,23,24]. PHQ-9 severity categories were minimal (0–4), mild (5–9), moderate (10–14), moderately severe (15–19) and severe (20–27) [22]. For the HDRS-17, a cut-off of ≥8 points was used, with severity categories of no depression (0–7), mild (8–16), moderate (17–23) and severe (≥24) depression [25,26,27]. Validated German versions are available for both instruments [24,27]. Since the HDRS cut-off already includes mild depressive symptoms, the two definitions are not entirely equivalent; we therefore conducted sensitivity analyses limited to the Homburg cohort (see Section 2.3). Data on psychopharmacological medication were available for the Mainz cohort only. In Homburg, patients with a known psychiatric diagnosis were not approached.

### 2.1. Assessment of Health Literacy

As in our previous studies, HL was assessed with the HLQ [13,14]. The German version of this instrument was validated in 2017 [5,28]. The questionnaire consists of 44 items grouped into nine distinct HL domains [5]. The five scales of Part I use a 4-point response format (from strongly disagree to strongly agree), whereas the four scales of Part II use a 5-point format reflecting how difficult a given task is perceived to be (from cannot do/always difficult to always easy). For all scales, higher values reflect better HL.

The nine HLQ scales are as follows:

1. Feeling understood and supported by healthcare providers (HPS) (4 items);
2. Having sufficient information to manage my health (HSI) (4 items);
3. Actively managing my health (AMH) (5 items);
4. Social support for health (SS) (5 items);
5. Appraisal of health information (CA) (5 items);
6. Ability to actively engage with healthcare providers (AE) (5 items);
7. Navigating the healthcare system (NHS) (6 items);
8. Ability to find good health information (FHI) (5 items);
9. Understanding health information well enough to know what to do (UHI) (5 items).

As recommended by the developers, each of the nine scales was analyzed on its own, and no total score was computed [5]. The scales differ in their items, in the tasks they address and in their response formats (Part I: 1–4; Part II: 1–5), so that scores cannot be compared directly across scales. In addition, there are no evidence-based thresholds for dichotomizing HLQ scores.

Swinburne University (Hawthorn, Australia) granted permission to use the HLQ. Patients filled in the paper version of the questionnaire with the help of a trained healthcare professional.

### 2.2. Ethics

The study complied with the principles of the Declaration of Helsinki and was approved by the ethics committees of the Landesärztekammer Rheinland-Pfalz (2019-14483) and the Ärztekammer des Saarlandes (Kenn-Nr. 61/24). All participants gave written informed consent.

### 2.3. Statistical Analysis

Continuous variables are reported as medians with interquartile ranges (IQR), and categorical variables as absolute numbers with percentages. Continuous variables were compared between two groups with the Mann–Whitney U test, and categorical variables with the χ^2^ test or Fisher’s exact test, depending on expected cell frequencies. Because univariable comparisons of HL may be confounded by differences in age, sex or education, we additionally fitted multivariable linear regression models including sex, age, body mass index (BMI), schooling (>10 years) and ACLD and report standardized regression coefficients (β). The robustness of the results was examined in the following sensitivity analyses: (i) further adjustment for diabetes mellitus, arterial hypertension and chronic kidney disease; (ii) exclusion of patients with decompensated cirrhosis; (iii) restriction to the Homburg cohort, analyzing the PHQ-9 both as a dichotomous (≥10) and as a continuous variable, the latter being independent of any single cut-off, with the continuous model also fitted with the additional adjustment described in (i); (iv) use of LSM ≥ 10 kPa to define ACLD in the Homburg cohort; and (v) restriction to Homburg patients meeting strict MASLD criteria (≥1 cardiometabolic risk factor and AUDIT score < 8), with the PHQ-9 analyzed both as a dichotomous and as a continuous variable. HLQ scale scores were computed as the mean of the corresponding items following the developers’ scoring instructions and were cross-checked against the item-level data; patients with implausible scale scores that could not be verified at item level were excluded (Supplementary Figure S1). Since all eligible patients recruited during the study period were included in this exploratory analysis, no a priori sample size calculation was carried out. According to a sensitivity power analysis, the comparison of 68 with 166 patients provided 80% power (two-sided α = 0.05) to detect group differences with an effect size of Cohen’s d ≥ 0.41 and, in the multivariable models, an effect of a single predictor of f^2^ ≥ 0.034. Given the exploratory character of all analyses, *p* values were not adjusted for multiple comparisons. A two-sided *p* value < 0.05 was considered statistically significant unless stated otherwise; in view of the number of tests, *p* values should be interpreted cautiously. All analyses were performed with IBM SPSS Statistics, version 27.0 (IBM Corp., Armonk, NY, USA).

## 3. Results

### 3.1. Baseline

The cohort comprised 234 patients with SLD (Homburg *n* = 209; Mainz *n* = 25; Supplementary Figure S1), of whom 68 (29%) exhibited symptoms of depression (Table 1). The prevalence of depressive symptoms was 25.8% (54/209) in Homburg and 56.0% (14/25) in Mainz. The median age was comparable between patients with and without depressive symptoms (55 (40; 61) vs. 55 (43; 65) years). Men accounted for 41% of patients with and 52% of patients without depressive symptoms (*p* = 0.16). The proportion of patients with more than 10 years of schooling was similar in both groups (87% vs. 86%). Median liver stiffness was similar (8.1 (6.1; 12.2) vs. 8 (5.7; 15) kPa). The prevalence of diabetes mellitus, arterial hypertension, chronic kidney disease and ACLD did not differ significantly between the groups (Table 1).

With regard to the etiology of SLD, all 208 Homburg patients with available CAP had values ≥ 275 dB/m (median 318 (294; 340) dB/m), 205 of 209 (98%) fulfilled at least one cardiometabolic criterion of MASLD, and none of the 173 patients with documented daily alcohol intake reported consumption ≥ 20/30 g/day (women/men); thus, no patient met the criteria for MetALD or alcohol-related liver disease. Daily alcohol intake in grams was not recorded in 36 patients; however, the AUDIT was available in 208 of 209 patients, including all of these 36 patients (median AUDIT score 1; AUDIT ≥ 8 in 3 of them). Overall, 15 patients (7%) had an AUDIT score ≥ 8, and 4 patients had no documented cardiometabolic risk factor. All 25 patients from Mainz had MASH-related cirrhosis; in this cohort, alcohol consumption within the preceding two weeks was recorded (reported by 1 of 25 patients), and alcohol-related liver disease had been excluded by the treating hepatologists. Of the Mainz patients, 17 (68%) had decompensated disease (Supplementary Table S1). In Homburg, LSM was <10 kPa in 137, 10–15 kPa in 22 and ≥15 kPa in 48 patients (missing in 2).

In Homburg, the median PHQ-9 score was 6 (3; 10); 85 patients (41%) had minimal, 70 (33%) mild, 40 (19%) moderate, 12 (6%) moderately severe and 2 (1%) severe depressive symptoms. In Mainz, the median HDRS-17 score was 10 (5; 13); 11 patients (44%) had no, 13 (52%) mild and 1 (4%) moderate depressive symptoms, and none had severe depressive symptoms (Supplementary Table S2).

As scales 1–5 (Part I) are scored from 1 to 4 and scales 6–9 (Part II) from 1 to 5, scale scores were compared only within each part. Within Part I, “Actively managing my health” (Scale 3) and “Appraisal of health information” (Scale 5) had the lowest scores in patients both with and without depressive symptoms (Table 1). Within Part II, median scores were similar across scales (range 3.5–4.0).

ACLD, advanced chronic liver disease (LSM ≥ 15 kPa in Homburg; all Mainz patients had MASH-related cirrhosis); BMI, body mass index; HDRS-17, 17-item Hamilton Depression Rating Scale; IQR, interquartile range; LSM, liver stiffness measurement; MASH, metabolic dysfunction-associated steatohepatitis; PHQ-9, Patient Health Questionnaire-9; SLD, steatotic liver disease; Scale 1: Feeling understood and supported by healthcare providers, Scale 2: Having sufficient information to manage my health, Scale 3: Actively managing my health, Scale 4: Social support for health, Scale 5: Appraisal of health information, Scale 6: Ability to actively engage with healthcare providers, Scale 7: Navigating the healthcare system, Scale 8: Ability to find good health information, and Scale 9: Understanding health information well enough to know what to do.

### 3.2. Unadjusted Comparison of Health Literacy Between SLD Patients with and Without Depressive Symptoms

**Figure 1:**
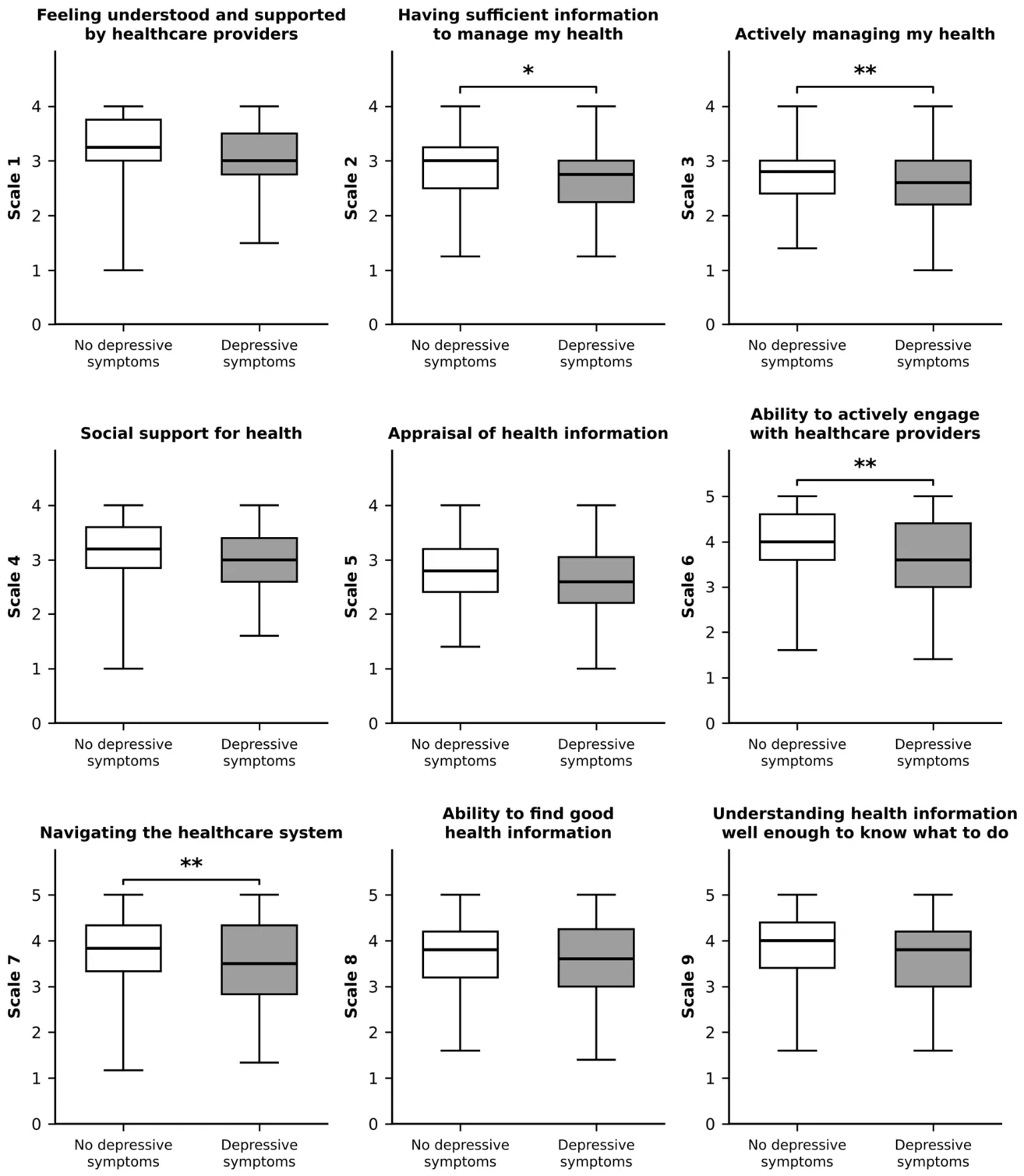
Differences in HLQ subscale scores between patients with and without depressive symptoms (*n* = 234; depressive symptoms defined as PHQ-9 ≥ 10 in Homburg or HDRS-17 ≥ 8 in Mainz). Boxes indicate median and IQR, whiskers minimum and maximum. Mann–Whitney U test: * *p* < 0.05, ** *p* < 0.01, not significant when not indicated.

Univariable comparisons of the HLQ scale scores between patients with and without depressive symptoms are shown in Figure 1. Patients with depressive symptoms had significantly lower scores on scale 2 (“Having sufficient information to manage my health”, *p* = 0.01), scale 3 (“Actively managing my health”, *p* = 0.003), scale 6 (“Ability to actively engage with healthcare providers”, *p* = 0.003) and scale 7 (“Navigating the healthcare system”, *p* = 0.007). The two groups did not differ significantly on scale 1 (“Feeling understood and supported by healthcare providers”), scale 4 (“Social support for health”), scale 5 (“Appraisal of health information”), scale 8 (“Ability to find good health information”) or scale 9 (“Understanding health information well enough to know what to do”).

### 3.3. Unadjusted Comparison of Health Literacy Between SLD Patients with and Without cACLD in the Homburg Cohort

**Figure 2:**
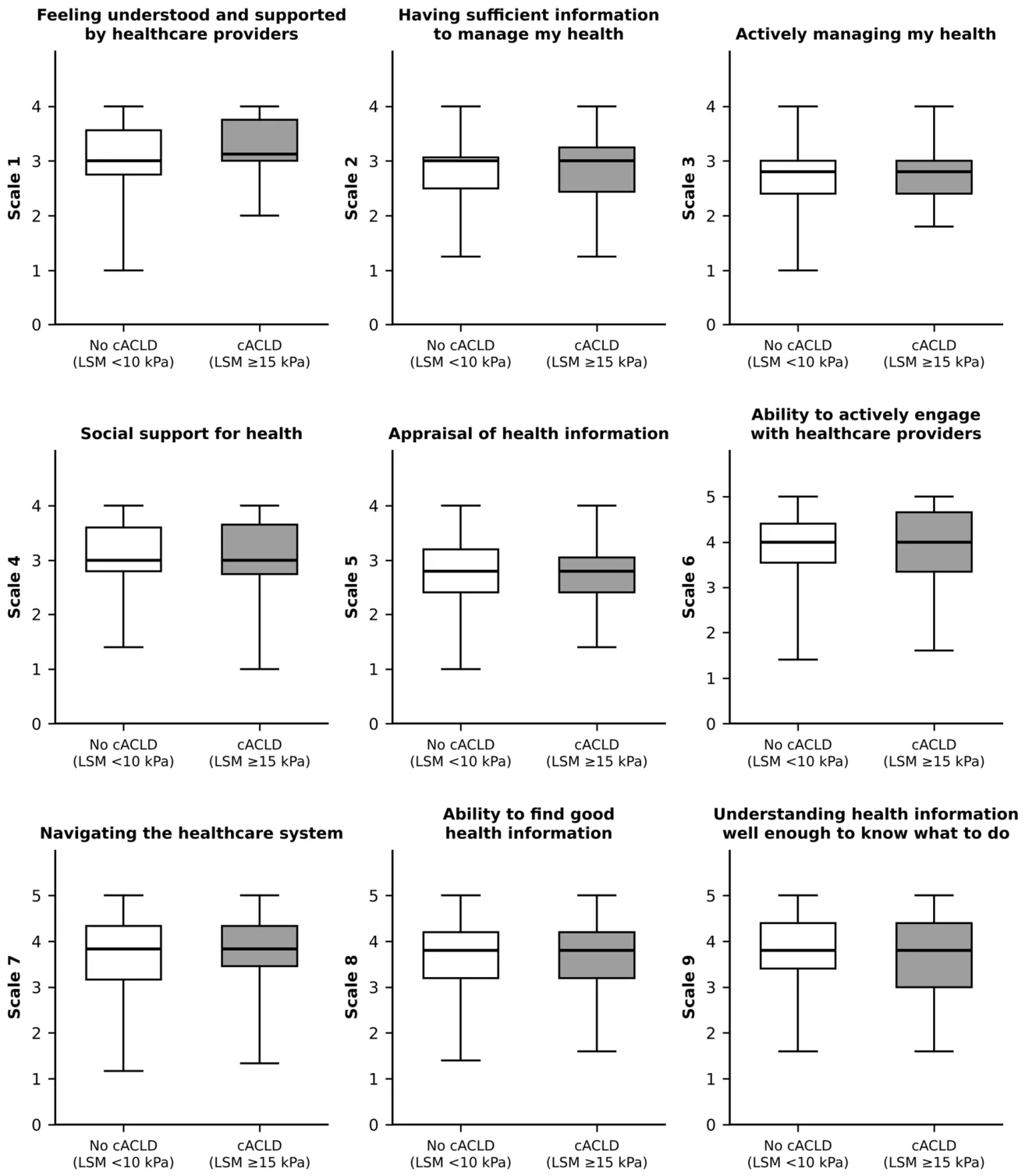
Differences in health literacy questionnaire (HLQ) scale scores between patients in the Homburg cohort with cACLD (LSM ≥ 15 kPa, *n* = 48) and without cACLD (LSM < 10 kPa, *n* = 137). Patients with LSM of 10–15 kPa or missing LSM were not included. Boxes indicate median and IQR, whiskers minimum and maximum. Mann–Whitney U test; no significant differences were observed.

We have previously reported that patients with liver cirrhosis are at increased risk of low HL [13]. To examine the role of liver disease stage in a homogeneous population without decompensation, Homburg patients were divided into those with cACLD (LSM ≥ 15 kPa; *n* = 48) and those in whom cACLD was excluded (LSM < 10 kPa; *n* = 137) according to the Baveno VII and VIII criteria [20,21]. Patients with LSM in the grey zone (10–15 kPa; *n* = 22) or without LSM (*n* = 2) were not part of this comparison; the Mainz patients with MASH-related cirrhosis, the majority of whom were decompensated, are characterized separately (Supplementary Table S1). None of the nine HLQ scales differed significantly between the groups (all *p* > 0.4), indicating that the stage of liver disease may have little influence on HL in patients with SLD (Figure 2).

### 3.4. Adjusted Comparison of Health Literacy Between SLD Patients with and Without Depressive Symptoms

To adjust for potential confounding by sex, age, BMI, education (more than 10 years of schooling) and ACLD, we compared SLD patients with and without depressive symptoms in multivariable linear regression models for each of the nine HLQ scales (Table 2). Depressive symptoms remained independently associated with lower scores on scale 2 (“having sufficient information to manage my health”, β = −0.14, *p* = 0.04), scale 3 (“actively managing my health”, β = −0.15, *p* = 0.02), scale 6 (“ability to actively engage with healthcare providers”, β = −0.21, *p* = 0.003) and scale 7 (“navigating the healthcare system”, β = −0.16, *p* = 0.02), but not on scale 9 (β = −0.11, *p* = 0.09). A higher BMI was linked to lower scores on scales 3, 4, 5, 6 and 7, whereas neither age nor ACLD showed an independent association with any HLQ scale.

### 3.5. Sensitivity Analyses

The results of the sensitivity analyses are summarized in Table 3. Additional adjustment for diabetes mellitus, arterial hypertension and chronic kidney disease did not materially change the associations (scale 6: β = −0.20, *p* = 0.004; scale 7: β = −0.16, *p* = 0.02). After exclusion of the 17 patients with decompensated cirrhosis, the associations with scales 2, 3 and 6 remained significant (scale 6: β = −0.17, *p* = 0.02), whereas the association with scale 7 was of borderline significance (β = −0.14, *p* = 0.053). In the analysis restricted to the Homburg cohort (*n* = 209), depressive symptoms defined as PHQ-9 ≥ 10 remained associated with lower scores on scale 3 (β = −0.16, *p* = 0.02) and scale 6 (β = −0.16, *p* = 0.03), whereas the association with scale 7 did not reach statistical significance (β = −0.11, *p* = 0.11). When the PHQ-9 was analyzed as a continuous variable, higher PHQ-9 scores were associated with lower scores on scales 2, 3, 6 and 7 (scale 6: β = −0.20, *p* = 0.008; scale 7: β = −0.15, *p* = 0.04). Results were similar when ACLD was defined as LSM ≥ 10 kPa and when the analysis was restricted to the 189 Homburg patients fulfilling strict MASLD criteria. Overall, the association between depressive symptoms and lower scores on scale 6 (“Ability to actively engage with healthcare providers”) was consistent across all sensitivity analyses.

## 4. Discussion

Using the HLQ, this study provides a domain-specific assessment of HL in patients with SLD. Many of these patients have metabolic syndrome with its numerous clinical complications [17]. In addition to somatic disease, mental comorbidities are common in this population and may affect HL [18,29]. We found that depressive symptoms were consistently linked to lower scores in particular HL domains, which may help clinicians to recognize patients in this population who are at risk of low HL. Specifically, patients with SLD and depressive symptoms scored lower on HLQ scales 2 (Having sufficient information to manage my health), 3 (Actively managing my health), 6 (Ability to actively engage with healthcare providers) and 7 (Navigating the healthcare system). These associations persisted after adjustment for age, sex, BMI, educational level and ACLD, and the association with scale 6 was stable in every sensitivity analysis, including the analyses confined to the Homburg cohort and those using the PHQ-9 as a continuous variable.

Importantly, the HLQ captures patients’ self-reported perceptions of their abilities rather than objectively observed behavior. Moreover, no validated cut-off values defining limited HL are available for the HLQ; HLQ scores therefore reflect relative differences between groups rather than a categorical classification of low HL [5]. Depression is characterized by negative appraisals of oneself, low self-efficacy, reduced energy and loss of motivation, all of which may lead affected patients to rate their capacity to engage with healthcare providers or to navigate the healthcare system less favorably, even if their actual behavior does not differ. Lower HLQ scores in patients with depressive symptoms should therefore not be interpreted as evidence of impaired healthcare behavior, but rather as reduced perceived capability and confidence in these domains. At the same time, perceived capability is clinically relevant in itself, as it may influence help-seeking, adherence and participation in shared decision-making. Whether lower HLQ scores in patients with depressive symptoms translate into differences in observed healthcare utilization or clinical outcomes should be addressed in longitudinal studies combining self-reported and objective measures. Although HL is frequently neglected, it is a key element of chronic disease management and should be embedded in best-practice care in order to improve long-term outcomes [30,31].

A high prevalence of depression in patients with MASLD has also been reported in a large Asian cohort [29]. Consistent with this, 29% of our patients had depressive symptoms, underlining that depression is a frequent comorbidity in SLD that may adversely affect HL. Low HL also appears to be frequent in SLD independent of depressive symptoms: in a diverse cohort of 151 patients with SLD, Figueroa et al. found low HL in almost 70% of participants [32]. A direct comparison with our data is, however, limited, because that study used Pfizer’s Newest Vital Sign (NVS) [33], a six-item screening tool that mainly captures functional reading and numeracy and thus only a narrow range of skills; moreover, the NVS has not been validated in German-speaking populations. The HLQ, with its nine separate scales, allows a considerably more differentiated and comprehensive evaluation of HL [5].

From a clinical perspective, these findings are relevant because the management of SLD relies predominantly on sustained lifestyle modification, including dietary changes, weight reduction and physical activity, as well as on long-term adherence to surveillance and to the treatment of cardiometabolic comorbidities. The HLQ domains affected in patients with depressive symptoms—having sufficient information, actively managing one’s health, engaging with healthcare providers and navigating the healthcare system—are prerequisites for these tasks. Patients with SLD and depressive symptoms may therefore require additional support: for example, structured and repeated information, shared goal-setting, involvement of dietitians and mental health professionals within multidisciplinary care models, and active coordination of appointments and referrals. Systematic screening for depressive symptoms with brief instruments such as the PHQ-9 could help to identify these patients in hepatology outpatient clinics, and the treatment of depression may represent an additional lever to improve engagement in lifestyle interventions. Whether such approaches improve HL and clinical outcomes needs to be evaluated prospectively.

Earlier work has shown that advanced liver disease is associated with lower HL and represents a stronger risk factor for low HL than other gastrointestinal conditions such as gastrointestinal cancer or inflammatory bowel disease [13,14]. We therefore stratified our patients with SLD by the presence of cACLD as determined by LSM. Non-invasive tests, and LSM in particular, have become the clinical standard for staging liver fibrosis. They quantify liver stiffness reliably, discriminate well for significant fibrosis and cirrhosis, can additionally quantify hepatic steatosis and, being non-invasive, have replaced routine liver biopsy in many settings [34]. In our Homburg cohort, HLQ scores did not differ between patients with and without cACLD, and ACLD showed no independent association with any HLQ scale in the multivariable models. This suggests that the stage of liver disease itself is not a major independent determinant of HL in SLD. By contrast, a higher BMI was independently related to lower scores on five of the nine scales (scales 3–7), pointing to BMI as a more relevant marker of patients with SLD at risk of low HL. Nevertheless, BMI and age seem to be general risk factors for low HL, as comparable associations have been described in healthy populations and in patients with other diseases. In a Taiwanese cohort of 1010 adults attending health check-ups, a higher BMI was associated with significantly lower HL [35]. Likewise, a population-based survey of 162,209 Taiwanese sixth-grade pupils (11–12 years) revealed a close relationship between HL and weight status: obesity was less prevalent among children in the highest than in the lowest HL quartile (12.4% vs. 17.4%), and after adjustment for sex, ethnicity, self-rated health and health behaviors, higher HL remained independently associated with a lower likelihood of obesity (relative risk ratio [RRR] = 0.94, *p* < 0.001) and of underweight (RRR = 0.83, *p* < 0.001) [36]. A cross-sectional study in children from the United States reported a similar relationship, indicating that this association is not restricted to Asian populations [37]. In the general population, age seems to be even more closely related to HL than BMI. Both large cross-sectional surveys and meta-analyses show a consistent inverse relationship, with limited HL being considerably more frequent in older than in younger adults. A meta-analysis of 29 studies, for instance, found a strong association between older age and limited HL when HL was measured by tests of reading comprehension, reasoning and numeracy (random-effects odds ratio [OR] = 4.20; 95% confidence interval [CI]: 3.13–5.64), whereas the association was much weaker when HL was measured mainly by medical vocabulary (random-effects OR = 1.19; 95% CI: 1.03–1.37) [38]. Similarly, in a German cross-sectional survey of about 2000 respondents, the proportion with limited perceived HL rose with age, from 47.3% at 15–29 years and 47.2% at 30–45 years to 55.2% at 46–64 years and 66.4% at 65 years and older; in all age groups, limited perceived HL went along with limited functional HL, lower social status and more frequent physician visits [39]. However, none of these studies used the HLQ, which limits comparability with our findings; in the present study, age was not independently associated with any HLQ scale after multivariable adjustment.

Several limitations need to be considered. First, owing to the cross-sectional design, associations between the study variables and low HL can be identified, but causal conclusions cannot be drawn. It therefore remains open whether depressive symptoms lead to lower HL or whether limited HL makes patients more susceptible to depressive symptoms; longitudinal studies are needed to clarify this. Second, all analyses were exploratory, without correction for multiple testing and without an a priori sample size calculation, so that smaller effects may have gone undetected. The results should therefore be interpreted cautiously and require confirmation in larger cohorts. Third, depressive symptoms were measured with two different instruments, the clinician-rated HDRS in Mainz and the self-administered PHQ-9 in Homburg, whose cut-offs are not fully equivalent, since an HDRS score ≥ 8 already includes mild symptoms. This probably explains, at least in part, the higher prevalence of depressive symptoms in Mainz (56.0% vs. 25.8%). Moreover, the Mainz cohort comprised only patients with MASH-related cirrhosis, the majority of whom had decompensated disease. We addressed this heterogeneity with sensitivity analyses restricted to the Homburg cohort and excluding decompensated patients, which yielded consistent results for scale 6. In addition, although both instruments are well suited for screening, a diagnosis of depression can only be established by a trained professional. Fourth, data on psychopharmacological treatment were available only for the Mainz cohort, and comorbidity data were limited to diabetes mellitus, arterial hypertension and chronic kidney disease, so that residual confounding cannot be ruled out. Fifth, as the HLQ is based on self-report, depressive symptoms may have biased patients’ assessment of their own health-related abilities. Finally, other mental disorders such as anxiety disorders were not assessed, and their additional influence on HL cannot be excluded.

## 5. Conclusions

In summary, this bicentric study demonstrates that, in patients with SLD, depressive symptoms are linked to lower self-reported HL in specific domains, most notably the perceived ability to engage with healthcare providers and to find, access and coordinate appropriate healthcare services. These results may help clinicians to recognize patients with SLD who are at increased risk of low HL and to adapt supportive measures to their needs.

## Figures and Tables

**Table 1:** Baseline characteristics of patients with steatotic liver disease with and without depressive symptoms.

Variable	Without Depressive Symptoms	With Depressive Symptoms	Total	*p* Value
*n*	166	68	234	
Homburg/Mainz, *n*	155/11	54/14	209/25	0.004
Age, years, median (IQR)	55 (43; 65)	55 (40; 61)	55 (41; 64)	0.27
BMI, kg/m^2^, median (IQR)	31.6 (28.4; 39.6)	34.3 (29.2; 45.7)	32.3 (28.7; 41.1)	0.08
Liver stiffness, kPa *, median (IQR)	8 (5.7; 15)	8.1 (6.1; 12.2)	8 (5.7; 13.4)	0.80
Male sex, *n* (%)	87 (52)	28 (41)	115 (49)	0.16
Schooling > 10 years, *n* (%)	140 (86)	58 (87)	198 (86)	1.00
Diabetes mellitus, *n* (%)	46 (28)	26 (39)	72 (31)	0.15
Arterial hypertension, *n* (%)	86 (52)	36 (54)	122 (53)	0.97
Chronic kidney disease, *n* (%)	13 (8)	5 (7)	18 (8)	1.00
ACLD (LSM ≥ 15 kPa or established cirrhosis), *n* (%)	50 (30)	23 (34)	73 (31)	0.66
**Health Literacy Questionnaire, median (IQR)**				
Scale 1	3.25 (3; 3.75)	3 (2.75; 3.5)	3 (2.75; 3.75)	0.06
Scale 2	3 (2.5; 3.25)	2.75 (2.25; 3)	3 (2.5; 3.25)	0.01
Scale 3	2.8 (2.4; 3)	2.6 (2.2; 3)	2.8 (2.4; 3)	0.003
Scale 4	3.2 (2.85; 3.6)	3 (2.6; 3.4)	3.1 (2.8; 3.6)	0.06
Scale 5	2.8 (2.4; 3.2)	2.6 (2.2; 3.05)	2.8 (2.4; 3.2)	0.14
Scale 6	4 (3.6; 4.6)	3.6 (3; 4.4)	4 (3.4; 4.4)	0.003
Scale 7	3.83 (3.33; 4.33)	3.5 (2.83; 4.33)	3.83 (3.17; 4.33)	0.007
Scale 8	3.8 (3.2; 4.2)	3.6 (3; 4.25)	3.8 (3.2; 4.2)	0.14
Scale 9	4 (3.4; 4.4)	3.8 (3; 4.2)	3.8 (3.4; 4.4)	0.09

* LSM was available only for the Homburg cohort (*n* = 207). *p* values were calculated using the Mann–Whitney U test or the χ^2^ or Fisher’s exact test, as appropriate. Depressive symptoms were defined as PHQ-9 ≥ 10 (Homburg) or HDRS-17 ≥ 8 (Mainz). Percentages refer to patients with available data.

**Table 2:** Multivariable linear regression analyses of health literacy questionnaire (HLQ) scales with depressive symptoms in the pooled cohort (*n* = 234). Standardized regression coefficients (β) are shown.

	β	*p* Value		β	*p* Value
**Scale 1: Feeling understood and supported by healthcare providers (*n* = 227)**			**Scale 6: Ability to actively engage with healthcare providers (*n* = 213)**		
Depressive symptoms	−0.08	0.22	Depressive symptoms	−0.21	0.003
Sex (0 women, 1 men)	0.00	1.00	Sex (0 women, 1 men)	−0.08	0.24
Age	0.10	0.21	Age	−0.07	0.36
BMI	−0.09	0.23	BMI	−0.17	0.02
Schooling > 10 years	−0.02	0.78	Schooling > 10 years	0.11	0.11
ACLD	0.03	0.69	ACLD	−0.05	0.45
**Scale 2: Having sufficient information to manage my health (*n* = 227)**			**Scale 7: Navigating the healthcare system (*n* = 226)**		
Depressive symptoms	−0.14	0.04	Depressive symptoms	−0.16	0.02
Sex (0 women, 1 men)	−0.08	0.25	Sex (0 women, 1 men)	−0.09	0.17
Age	0.05	0.52	Age	−0.07	0.37
BMI	−0.12	0.13	BMI	−0.15	0.048
Schooling > 10 years	0.04	0.60	Schooling > 10 years	0.10	0.12
ACLD	−0.01	0.93	ACLD	−0.04	0.57
**Scale 3: Actively managing my health (*n* = 227)**			**Scale 8: Ability to find good health information (*n* = 226)**		
Depressive symptoms	−0.15	0.02	Depressive symptoms	−0.09	0.17
Sex (0 women, 1 men)	−0.02	0.80	Sex (0 women, 1 men)	−0.14	0.044
Age	0.14	0.052	Age	−0.09	0.27
BMI	−0.25	<0.001	BMI	−0.06	0.41
Schooling > 10 years	−0.06	0.39	Schooling > 10 years	0.14	0.047
ACLD	0.00	0.97	ACLD	−0.06	0.41
**Scale 4: Social support for health (*n* = 228)**			**Scale 9: Understanding health information well enough to know what to do (*n* = 226)**		
Depressive symptoms	−0.07	0.26	Depressive symptoms	−0.11	0.09
Sex (0 women, 1 men)	−0.04	0.59	Sex (0 women, 1 men)	−0.13	0.07
Age	−0.09	0.21	Age	−0.13	0.10
BMI	−0.28	<0.001	BMI	−0.12	0.11
Schooling > 10 years	−0.07	0.30	Schooling > 10 years	0.16	0.02
ACLD	0.04	0.53	ACLD	−0.11	0.11
**Scale 5: Appraisal of health information (*n* = 228)**					
Depressive symptoms	−0.05	0.47			
Sex (0 women, 1 men)	−0.02	0.76			
Age	0.01	0.92			
BMI	−0.15	0.049			
Schooling > 10 years	0.08	0.24			
ACLD	0.00	0.96			

ACLD, advanced chronic liver disease (LSM ≥ 15 kPa in Homburg or MASH-related cirrhosis in Mainz); BMI, body mass index; HDRS-17, 17-item Hamilton Depression Rating Scale; LSM, liver stiffness measurement; MASH, metabolic dysfunction-associated steatohepatitis; PHQ-9, Patient Health Questionnaire-9. Depressive symptoms: PHQ-9 ≥ 10 (Homburg) or HDRS-17 ≥ 8 (Mainz).

**Table 3:** Sensitivity analyses: standardized regression coefficients (β) and *p* values for depressive symptoms in each HLQ scale. Unless stated otherwise, all models were adjusted for sex, age, BMI, schooling and ACLD.

Model	*n*	Scale 1	Scale 2	Scale 3	Scale 4	Scale 5	Scale 6	Scale 7	Scale 8	Scale 9
(1) Primary analysis: pooled cohort, PHQ-9 ≥ 10/HDRS-17 ≥ 8	213– 228	−0.08 (0.22)	−0.14 (0.04)	−0.15 (0.02)	−0.07 (0.26)	−0.05 (0.47)	−0.21 (0.003)	−0.16 (0.02)	−0.09 (0.17)	−0.11 (0.09)
(2) Pooled cohort + diabetes, hypertension, CKD	212– 227	−0.07 (0.30)	−0.14 (0.043)	−0.14 (0.03)	−0.05 (0.44)	−0.05 (0.48)	−0.20 (0.004)	−0.16 (0.02)	−0.09 (0.17)	−0.11 (0.10)
(3) Pooled cohort excluding decompensated cirrhosis	196– 211	−0.06 (0.43)	−0.14 (0.043)	−0.17 (0.009)	−0.09 (0.18)	−0.01 (0.89)	−0.17 (0.02)	−0.14 (0.053)	−0.07 (0.35)	−0.08 (0.28)
(4) Homburg only, PHQ-9 ≥ 10	188– 203	−0.06 (0.41)	−0.12 (0.08)	−0.16 (0.02)	−0.08 (0.26)	−0.01 (0.87)	−0.16 (0.03)	−0.11 (0.11)	−0.06 (0.39)	−0.09 (0.22)
(5) Homburg only, PHQ-9 continuous	188– 203	−0.09 (0.25)	−0.18 (0.01)	−0.19 (0.007)	−0.13 (0.07)	−0.07 (0.32)	−0.20 (0.008)	−0.15 (0.04)	−0.09 (0.24)	−0.11 (0.14)
(6) Homburg only, PHQ-9 continuous + diabetes, hypertension, CKD	187– 202	−0.08 (0.31)	−0.18 (0.01)	−0.17 (0.01)	−0.10 (0.15)	−0.07 (0.32)	−0.19 (0.01)	−0.14 (0.059)	−0.08 (0.26)	−0.09 (0.20)
(7) Homburg only, ACLD defined as LSM ≥ 10 kPa	188– 203	−0.06 (0.42)	−0.12 (0.09)	−0.16 (0.02)	−0.08 (0.27)	−0.01 (0.88)	−0.15 (0.04)	−0.11 (0.12)	−0.06 (0.43)	−0.08 (0.27)
(8) Homburg only, strict MASLD (≥1 CMRF, AUDIT < 8), PHQ-9 ≥ 10	170– 184	−0.11 (0.16)	−0.19 (0.01)	−0.18 (0.01)	−0.14 (0.06)	−0.04 (0.57)	−0.16 (0.046)	−0.12 (0.10)	−0.06 (0.44)	−0.08 (0.29)
(9) Homburg only, strict MASLD (≥1 CMRF, AUDIT < 8), PHQ-9 continuous	170– 184	−0.16 (0.045)	−0.26 (<0.001)	−0.20 (0.005)	−0.20 (0.007)	−0.12 (0.13)	−0.20 (0.01)	−0.15 (0.044)	−0.08 (0.28)	−0.10 (0.20)

Values are standardized β (*p* value). For models 1–4, 7 and 8, depressive symptoms were entered as a binary variable (PHQ-9 ≥ 10 or HDRS-17 ≥ 8); for models 5, 6 and 9, the PHQ-9 score was entered as a continuous variable. Decompensated cirrhosis: current or previous ascites, variceal bleeding and/or overt hepatic encephalopathy. Strict MASLD: ≥ 1 cardiometabolic risk factor and AUDIT score < 8. ACLD, advanced chronic liver disease; AUDIT, Alcohol Use Disorders Identification Test; BMI, body mass index; CKD, chronic kidney disease; CMRF, cardiometabolic risk factor; HDRS-17, 17-item Hamilton Depression Rating Scale; HLQ, Health Literacy Questionnaire; LSM, liver stiffness measurement; MASLD, metabolic dysfunction-associated steatotic liver disease; PHQ-9, Patient Health Questionnaire-9.

## Data Availability

The data presented in this study are available on request from the corresponding authors. The data are not publicly available due to privacy restrictions.

## Supplementary Materials

The following supporting information can be downloaded at: https://www.mdpi.com/article/10.3390/jcm15218474/s1, Figure S1: Patient flow. Of 241 patients with SLD enrolled in Homburg, 32 were excluded (HLQ not completed, n = 29; HLQ scale scores implausible and not verifiable against item-level questionnaire data, n = 2; PHQ-9 missing, n = 1). From the established Mainz cohort of 89 patients with cirrhosis who had completed the HLQ, the 25 patients with MASH-related cirrhosis were included.; Table S1: Baseline characteristics of patients with MASLD (MASH)-associated cirrhosis in the Mainz cohort, stratified by depressive symptoms (HDRS-17 ≥ 8); Table S2: Distribution of depression severity according to the PHQ-9 (Homburg) and HDRS-17 (Mainz).

## Abbreviations

ACLD	advanced chronic liver disease
AUDIT	Alcohol Use Disorders Identification Test
BMI	body mass index
cACLD	compensated advanced chronic liver disease
CAP	controlled attenuation parameter
CI	confidence interval
CKD	chronic kidney disease
CMRF	cardiometabolic risk factor
HDRS	Hamilton Depression Rating Scale
HDRS-17	17-item Hamilton Depression Rating Scale
HL	health literacy
HLQ	Health Literacy Questionnaire
IQR	interquartile range
LSM	liver stiffness measurement
MASH	metabolic dysfunction-associated steatohepatitis
MASLD	metabolic dysfunction-associated steatotic liver disease
MetALD	metabolic dysfunction and alcohol-associated liver disease
NASH	non-alcoholic steatohepatitis
NVS	Newest Vital Sign
OR	odds ratio
PHQ-9	Patient Health Questionnaire-9
RRR	relative risk ratio
SLD	steatotic liver disease
WHO	World Health Organization
