# Supplementary material for: Low Health Literacy in Patients with Steatotic Liver Disease and Depressive Symptoms
Source: J Clin Med. 2026 Oct 30;15(21):8474. doi: 10.3390/jcm15218474 (PMC13764474; doi:10.3390/jcm15218474)
Supplement: Supplementary file 1 [file jcm-15-08474-s001.zip › jcm-4637765-supplementary.pdf]

Supporting information

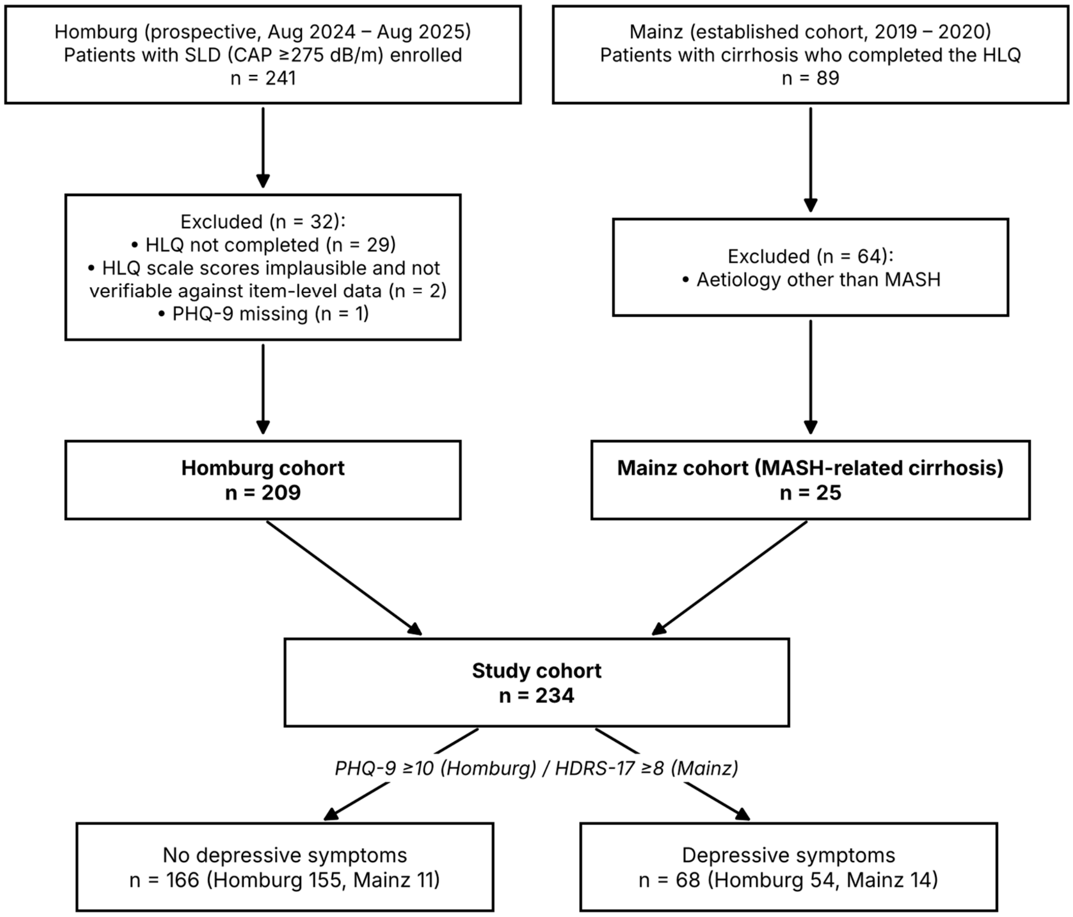

Figure S1: Patient flow. Of 241 patients with SLD enrolled in Homburg, 32 were excluded (HLQ not completed, n = 29; HLQ scale scores implausible and not verifiable against item-level questionnaire data, n = 2; PHQ-9 missing, n = 1). From the established Mainz cohort of 89 patients with cirrhosis who had completed the HLQ, the 25 patients with MASH-related cirrhosis were included.

Table S1: Baseline characteristics of patients with MASLD (MASH)-associated cirrhosis in the Mainz cohort, stratified by depressive symptoms (HDRS-17 ≥ 8)

Variable	No depressive symptoms	Depressive symptoms
n	11	14
Age, years, median (IQR)	67 (63; 71)	60 (58; 67)
Male sex, n (%)	7 (64)	7 (50)
BMI, kg/m², median (IQR)	28.1 (25; 30)	28.3 (25.8; 32.9)
Years of education, median (IQR)	13 (12; 16)	13 (12; 14)
Child-Pugh class A / B / C, n	8 / 3 / 0	5 / 7 / 2
MELD score, median (IQR)	9 (8; 12)	16 (10; 17)
Decompensated cirrhosis*, n (%)	7 (64)	10 (71)
Ascites (current or previous), n (%)	6 (55)	9 (64)
Previous variceal bleeding, n (%)	2 (18)	5 (36)
Previous overt hepatic encephalopathy, n (%)	0 (0)	4 (29)
Diabetes mellitus, n (%)	3 (27)	6 (43)
Psychopharmacological treatment, n (%)	0 (0)	3 (21)
HDRS-17 score, median (IQR)	4 (2; 6)	12 (10; 15)
**Health Literacy Questionnaire, median (IQR)**		

Scale 1	3.25 (3; 3.88)	3 (2.75; 3.75)
Scale 2	3 (2.5; 3)	2.88 (2.31; 3.19)
Scale 3	2.8 (2.6; 3)	2.8 (2.6; 3)
Scale 4	3.4 (2.9; 3.5)	3.2 (2.64; 3.75)
Scale 5	2.8 (2.7; 3.1)	2.6 (2.45; 2.95)
Scale 6	4 (3.4; 4)	3.5 (3.2; 3.8)
Scale 7	3.5 (3.17; 3.75)	3.33 (2.92; 3.67)
Scale 8	3.4 (2.8; 3.9)	3.4 (3; 3.8)
Scale 9	3.4 (3.1; 3.8)	3.5 (2.9; 3.8)

*Decompensation was defined according to Baveno VII/VIII as current or previous clinically evident ascites, variceal bleeding and/or overt hepatic encephalopathy. HDRS-17, 17-item Hamilton Depression Rating Scale; MELD, Model for End-Stage Liver Disease; IQR, interquartile range; Scale 1: Feeling understood and supported by healthcare providers, Scale 2: Having sufficient information to manage my health, Scale 3: Actively managing my health, Scale 4: Social support for health, Scale 5: Appraisal of health information, Scale 6: Ability to actively engage with healthcare providers, Scale 7: Navigating the healthcare system, Scale 8: Ability to find good health information, Scale 9: Understanding health information well enough to know what to do.

Table S2: Distribution of depression severity according to the PHQ-9 (Homburg) and the HDRS-17 (Mainz)

Instrument / severity category	Score range	n (%)
**PHQ-9, Homburg (n = 209); median (IQR) 6 (3; 10)**		
Minimal	0–4	85 (40.7)
Mild	5–9	70 (33.5)
Moderate †	10–14	40 (19.1)
Moderately severe †	15–19	12 (5.7)
Severe †	20–27	2 (1.0)
**HDRS-17, Mainz (n = 25); median (IQR) 10 (5; 13)**		
No depression	0–7	11 (44.0)
Mild †	8–16	13 (52.0)
Moderate †	17–23	1 (4.0)
Severe †	≥24	0 (0.0)

† Categories classified as depressive symptoms in the present analysis (PHQ-9 ≥ 10, n = 54; HDRS-17 ≥ 8, n = 14). Severity categories according to Kroenke et al. (PHQ-9) and Zimmerman et al. (HDRS-17). HDRS-17, 17-item Hamilton Depression Rating Scale; PHQ-9, Patient Health Questionnaire-9.
